# Solvent-free bulk polymerization of lignin-polycaprolactone (PCL) copolymer and its thermoplastic characteristics

**DOI:** 10.1038/s41598-019-43296-2

**Published:** 2019-05-07

**Authors:** In-Kyung Park, Hanna Sun, Sung-Hoon Kim, Youngjun Kim, Go Eun Kim, Youngkwan Lee, Taesung Kim, Hyouk Ryeol Choi, Jonghwan Suhr, Jae-Do Nam

**Affiliations:** 1 Department of Polymer Science and Engineering, Sungkyunkwan University, Suwon, 16419 Republic of Korea; 2 Department of Energy Science, Sungkyunkwan University, Suwon, 16419 Republic of Korea; 3 School of Chemical engineering, Sungkyunkwan University, Suwon, 16419 Republic of Korea; 4 School of Mechanical engineering, Sungkyunkwan University, Suwon, 16419 Republic of Korea

**Keywords:** Sustainability, Polymers

## Abstract

The pristine lignin molecules contain multiple reactive hydroxyl [OH] groups, some of which undergo limited polymerization depending on their configuration (aromatic or aliphatic) or conformation. The key issue in lignin-polymerization is to quantify the number of hydroxyl groups in the pristine molecules for subsequent activation to specific lignin-polymer chain lengths or degree of grafting. In this study, using ε-caprolactone (CL) as a reactive solvent, we successfully polymerized CL on the [OH] sites in the kraft lignin macromonomers (LM, M_w_ = 1,520 g mol^−1^), which resulted in a thermoplastic lignin-polycaprolactone (PCL) grafted copolymer. We found that the average number of [OH] groups in the LM was 15.3 groups mol^−1^, and further detected 40–71% of the [OH] groups in the CL bulk polymerization. The degree of polymerization of PCL grown on each [OH] site ranged between 7 and 26 depending on the reaction conditions ([CL]/[OH] and reaction-time) corresponding to 4,780 and 32,600 g mol^−1^ of PCL chains per a LM. The thermoplastic characteristics of the synthesized lignin-PCL copolymers were established by the melt viscosity exhibiting a shear-thinning behavior, e.g., 921 Pa.s at 180 °C. The thermal stability was remarkable providing a T_id_ (2% of weight loss) of 230 °C of the copolymers, compared with 69 °C for the pristine lignin.

## Introduction

Reliable and practical approaches to lignin utilization have been intensively investigated in recent years as an alternative to replace petroleum-based materials^1^. Lignin is the most abundant aromatic natural polymer, with a three-dimensionally cross-linked phenolic structure. It contributes to the strength and rigidity of vascular plants. In pulp industry, a tremendous amount of kraft lignin is produced as a by-product in the form of black liquor (worldwide ca. 30 million tons/year), almost all of which is burnt as fuel^2,3^. Although it is an attractive renewable resource with enormous potential, only a small fraction of lignin (<1%) is currently used not as a fuel but as a value-added material such as fillers or additives^4^.

Although lignin has a huge potential for use as a source of biomass, several critical issues remain to be resolved for large-scale usage. Lignin is thermosetting in nature: It decomposes under heating without melting. Thermoplastic characteristics of lignins ensures the development of various value-added polymer blends and composites for utilization in feedstock industry such as large volume production methods including injection molding and extrusion^5,6^. However, the poor thermal stability and hydrophilic nature of lignin limits its use only in water-based and room-temperature (less than 50 °C) applications. The pristine lignin is hardly miscible with most organic solvents or polymers, and starts burning with odor and fumes upon heating over 50 °C, due to the presence of lightly-oxidative hydroxyl and methoxyl groups in the macromonomer chains^7–10^. Particularly, the oxidative and hydrophilic properties of the hydroxyl group in the lignin macromonomers (LMs) leads to poor compatibility with most synthetic polymers and results in aggregation to form large clusters of particles due to strong hydrogen bonding^9,10^.

Accordingly, the hydroxyl groups in lignin may be replaced or eliminated by various types of organic modification^5,10,11^ and polymerization techniques^5,6,12,13^. The reactivity of the two types of [OH] groups existing either in the phenolic or aliphatic linkages varies with the configuration and/or conformation of LMs, which interferes with their degree of polymerization and/or reaction kinetics^12,14,15^. Recently, we reported that ε-caprolactone (CL) and LMs react in the presence of DMAc^15^. In the reaction between CL and LMs, the hydroxyl groups in lignin are replaced by the reactive and controllable hydroxide groups. The CL can grow further as a polycaprolactone (PCL) via the CL ring-opening polymerization (ROP). The resulting lignin-PCL copolymers displays thermoplastic characteristics due to the grafted PCL chains in the LMs. One of the key issues involved in this route is the control of the degree of polymerization (DP) of grafted PCL in LMs. Another significant issue is quantifying the amount of [OH] in the LMs that selectively participate in the reaction with CL.

Furthermore, almost all the lignin modification reactions have been carried out in the presence of solvents^5,6,10–13,16,17^. When solvents are used in the lignin reactions, however, the products should be separated from unreacted reagents, catalysts, and solvents and washed through multiple sequential steps, which usually accompany environmental issues and cost incrementally. Accordingly, we believe that the most important progress in lignin research relates to bulk polymerization of lignin powders without using solvents. The reactant CL used in our study acts as a reactive solvent that disperses lignin powders at the initial stage of polymerization and CL is subsequently consumed as PCL. It may desirably allow bulk polymerization, which ensures a large-scale production of lignin-based polymer.

PCL is a biodegradable aliphatic-polyester plastic, with low melt viscosity, and facilitates the melting processes^18–20^. PCL has attracted significant attention because of its miscibility with other polymers, relative cost-effectiveness and suitability for polymer grafting and self-polymerization^19^. The high molecular polyesters have been prepared from oligomeric polyester precursors via ring-opening polymerization^21^. The grafted PCL chains can be used to modulate the physical and mechanical properties to induce biodegradability of the resulting polymers^21^. PCL has rubbery characteristics with an elongation at break of approximately 600%^22^, which can be used for toughening brittle lignin macromonomers.

In this study, we report a bulk polymerization of LM with CL to synthesize PCL-grafted lignin copolymers in the absence of any solvents. We analyzed the structure of the lignin-PCL copolymers using Nuclear Magnetic Resonance (NMR) spectroscopy, Gel Permeation Chromatograpy (GPC), and Fourie Transform Infrared (FT-IR) spectroscopy to measure the molecular weight and ester groups. The thermal stability and the melt viscosity of the resulting lignin-PCL copolymer were thoroughly investigated. This work provides a novel synthetic route for the preparation of lignin-based thermoplastic polymer, and demonstrates the conversion of lignin powder to a thermally-stable thermoplastic copolymer that can be blended with various synthetic polymers using extrusion and injection molding processes.

## Results and Discussion

### Preparation of lignin macromonomer for copolymerization

**Figure 1:**
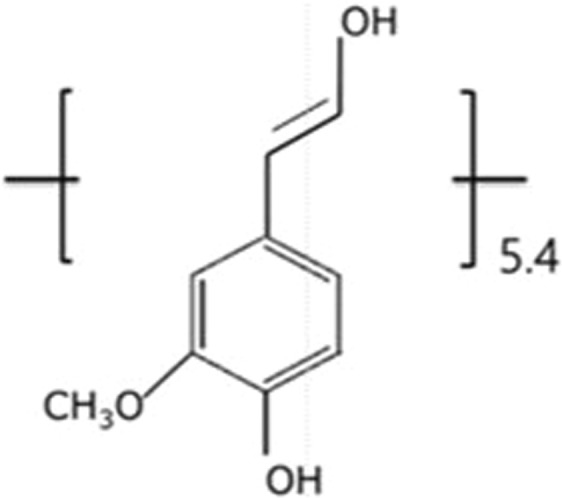
A model lignin macromonomer composed of 5.4 repeating monolignols (M_0_ = 180 gmol^−1^). The kraft lignin used in this study was M_n,LM_ = 970 and M_w,LM_ = 1,520 gmol^−1^.

The lignin structure varies greatly among plant species (softwood, hardwood, and non-wood like wheat straw) and it also changes according to the different extraction methods (kraft, sulfite, soda, organosolv, or steam explosion). It makes the chemical structure of LM is extremely complex^23^. Although lignin has not a regular structure, we can use the point to grow from monolignol to lignin macromer^24^. Among three commonly monolignol, coniferyl alcohol is the most predominat existing in the softwoods^25,26^. Due to quantifying the numerous [OH] groups, the LM is assumed to be a macropolyol repeating coniferyl aocohol (see Fig. 1).

The molecular weights of the LM are M_n,LM_ = 970 g mol^−1^, M_w,LM_ = 1520 g mol^−1^, and M_w,LM_/M_n,LM_ (MWD) ratio = 1.57. In this study, we assumed an LM composition of 5.4 repeating units (monolignols). The molecular weight of this monolignol was 180 g mol^−1^. The LM was polymerized with CL via ring-opening reaction, which was initiated by [OH] groups^27^. In this graft copolymerization, it is important to quantify the number of [OH] in each repeating unit of the pristine LM. The [OH] content of lignin is generally determined via spectroscopic techniques using NMR^28^. Compared with the direct NMR measurement of the pristine lignin, the acetylated lignin yielded better signal resolution, lower signal overlap, and proton coupling effects^7^. The lignin [OH] group is either aromatic or aliphatic, with different reactivity. Furthermore, the acetyl protons attached to aromatic or aliphatic chain were distinguished in NMR, which subsequently yielded different reactivity of [OH] in aromatic or aliphatic chains. As shown in the ^1^H-NMR spectra of our specimen in Fig. S1^10^, the acetyl protons of aliphatic and aromatic groups were 1.9 and 2.2 ppm, respectively. As discussed earlier, the average number of [OH] groups in the pristine LM was 15.3 corresponding to 10.09 mmol g^−1^, which yielded an aromatic [OH] content of 2.16 mmol g^−1^ and an aliphatic OH level of 7.93 mmol g^−1^. In other words, the [OH] in LM is composed of 21% of aromatic [OH] and 79% of aliphatic [OH]. Table 1 summarizes the molecular weight and analysis of [OH] using GPC and NMR. In order to increase the molecular weight of copolymers, we assumed that the degree of grafting and the length of grafted PCL chain were competitive. The efficiency of copolymerization is based on lignin [OH] content.

**Table 1:** Analysis of a lignin macromonomer (LM).

M_n,LM_ (g mol^−1^)^a^	M_w,LM_ (g mol^−1^)^a^	MWD (M_w,L_/M_n,L_)^a^	T_g_ (°C)^b^	Total OH (mmol g^−1^)	aromatic [OH] (mmol g^−1^)^c^	aliphatic [OH] (mmol g^−1^)^c^
970	1520	1.57	80	10.09	2.16	7.93

^a^Molecular weight and molecular weight distribution were measured by GPC with polystyrene standards. THF was used as eluent. ^b^The glass transition temperature of the pristine lignin is determined by differential scanning calorimetry (DSC). ^c^Calculated from ^1^H NMR spectra.

### Bulk polymerization of lignin-PCL copolymer

**Figure 2:**
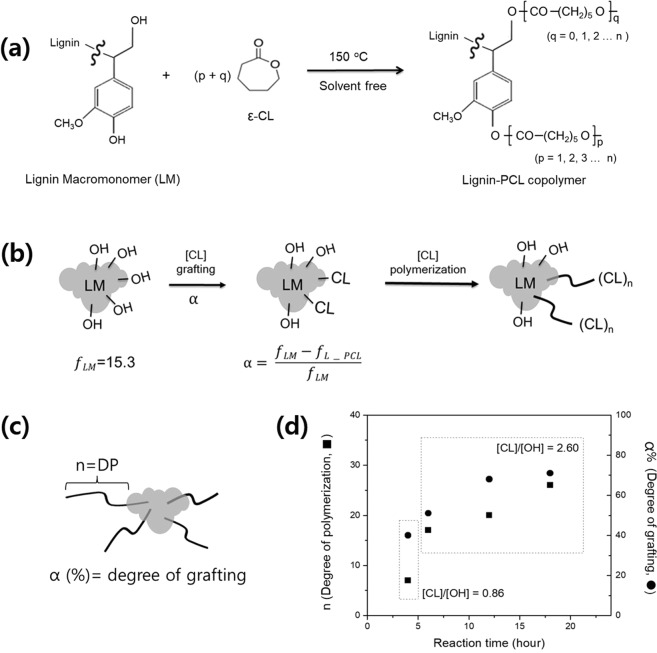
(**a**) Synthesis route of lignin-PCL copolymer using ring-opening polymerization of ε-caprolactone monomers on lignin in bulk. (**b**) Outline for the preparation of lignin-PCL copolymer. f_LM_ represents the functionality of a lignin macromonomer (LM), which was 15.3 in our kraft lignin. α is the degree of [CL] reaction based on [OH] functionality in a LM, indicating the degree of grafting. ‘*n*’ denotes the degree of polymerization in a PCL chain and was determined by ^1^H NMR. (**c**) General scheme of lignin-PCL copolymers with α (%) PCL chains. (**d**) Effect of chemical composition of LM on the structures of copolymers synthesized via ring-opening polymerization from bulk process.

The lignin-PCL copolymers were synthesized via ROP of CL used in bulk process (Fig. 2(a)). CL is initiated by [OH] groups in LM leads to graft copolymers which have extended PCL chains. ROP is a form of chain-growth polymerization, in which the terminal end of a polymer chain act as a reactive center where further cyclic monomers (CL) can react by opening its ring system and form a longer polymer chain^29^. As seen in Fig. 2(b), the functionality of a LM is indicated by *f*_*LM*_ = 15.3 and suggests the number of [OH] groups. The -COO- group is formed via opening of the CL ring, and reaction with the [OH] in lignin to form an ester linkage leaving -OH at the end of the CL chain, which subsequently reacts with CL monomers to grow a PCL. We considered another functionality of the lignin-PCL copolymer (*f*_*L_PCL*_) among the total [OH] groups in the reaction participating in the PCL reaction. As the PCL chains are grafted on the lignin [OH] sites, the degree of polymerization (DP, *n*) or the number of CL repeating units is represented in Fig. 2(b). Figure 2(c) shows a general scheme of lignin-PCL copolymers that PCL was grafted as a flexible segment to the LM in this study. PCL chains grow from the active [OH] sites in the LMs through ROP of CL monomers, and thus the degree of [CL] grafting (*α*) may be defined by the [OH] functionality in a LM as follows:

1 $${\rm{\alpha }}=\frac{{f}_{LM}-{f}_{L\_PCL}}{{f}_{LM}}$$

where *f*_*LM*_ is the functionality of a LM, which was 15.3 in our kraft lignin, and *f*_*L*-*PCL*_ represents the functionality [OH] of the lignin-PCL copolymer after grafting CL.

The bulk polymerization of lignin and CL can be controlled by measuring the torque of reacting mixture as a function of reaction time. Initially, CL monomers act like a solvent to dissolve the lignin powder and viscosity of copolymer is low. As the ROP of CL progresses, CLs are consumed eventually resulting in PCL. The molecular weight and the viscosity of copolymer increased. The torque of the copolymer is proportional to the viscosity, which increased with time as the polymerization progressed^30,31^. Most the viscosity of the reaction mixture increase dramatically with consuming monomers (CL). The composition ([CL]/[OH]) and the reaction time of the copolymers among the reaction parameters strongly affected the molecular weight.

**Figure 3:**
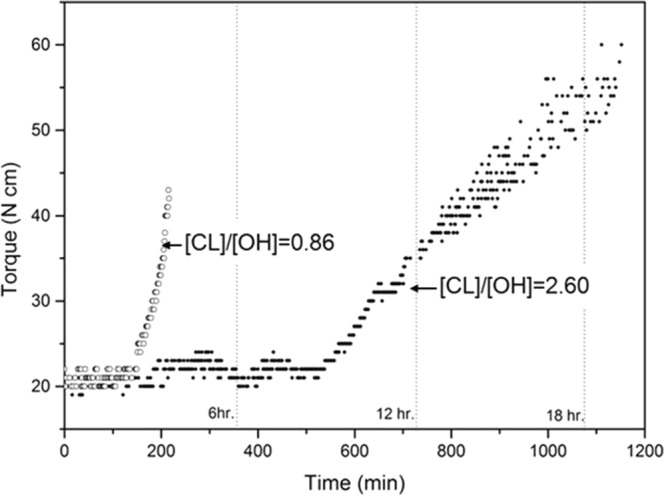
The changes in viscosity represented by the reaction torque as a function of time during bulk polymerization at 150 °C, when [CL]/[OH] molar ratio = 0.86 (◦, LigPCL11) and 2.60 (•, LigPCL13C).

Figure 3 displays the comparison of torque curves as a function of the bulk reaction time for two cases for visualizing the reaction clearly. At [CL]/[OH] = 0.86, [CL] monomer is slightly insufficent to [OH] of lignin, and this ratio has the minimum amount of CL to react with LM. The torque increased rapidly after 120 minutes, and the copolymerization occurred during 4 hours. After that the viscosity of copolymer no longer changed. It suggests that the total consumption of all the CL monomer due to low CL monomer levels was to restrict lengthening of the PCL chain or initiation of the [OH] groups in LM. In case of excessive [CL] levels at a [CL]/[OH] ratio of 2.6, the viscosity of copolymer increased gradually after 6 hours and bulk copolymerization occurred for 18 hours. Higher levels of CL monomer prolonged the reactio time. This ratio has the maximum amount of CL to copolymerize without any other solvent. It clearly suggests the progress of bulk copolymerization with respect to reaction time. In both cases, it is indicated that ths bulk process has the initiation and propagation stage including the grafting stages by change of viscositying the reaction progress clearly. At [CL]/[OH] = 0.86, [CL] monomer is slightly insufficent to [OH] of lignin, and it is the maximum amount of lignin that can be copolymerized in bulk process. The torque increased rapidly after 120 minutes, and the copolymerization occurred during 4 hours. After that the viscosity of copolymer no longer changed. It suggests that the total consumption of all the CL monomer due to low CL monomer levels was to restrict lengthening of the PCL chain or initiation of the [OH] groups in LM. In case of excessive [CL] levels at a [CL]/[OH] ratio of 2.6, the viscosity of copolymer increased gradually after 6 hours and bulk copolymerization occurred for 18 hours. Higher levels of CL monomer prolonged the reactio time. This ratio is adquate for achieving bulk polymerization with LM. It clearly suggests the progress of bulk copolymerization with respect to reaction time. In both cases, it is indicated that ths bulk process has the initiation and propagation stage including the graft stages by change of viscosity.

### Structural analysis of lignin-PCL copolymers

In designing the appropriate growth strategy for preparation of well- defined lignin-PCL copolymers from LM four groups with different reaction parameters was selected. As mentioned earlier, the copolymer samples organized with the two [CL]/[OH] ratios (0.86 and 2.60) and the reaction time from 6 to 18 hours at the same [CL]/[OH] = 2.60. As the [CL]/[OH] ratio increases from 0.86 (LigPCL11) to 2.60 (LigPCL13C), the resulting copolymer has 4 and 18 hours with the reaction time, 6,380 and 33,570 gmol^−1^ with M_w_. When the copolymerization time is changed for 6, 12, and 18 hours at [CL]/[OH] = 2.60, the resulting copolymer changes the larger molecular weight and the broader MWD. The molecular weight of LigPCL13A, LigPCL13B, and LigPCL13C increases 16,520 gmol^−1^, 25,360 gmol^−1^, and 33,570 gmol^−1^, respectively. The change of the composition led to more noticeable than that of the reaction time. These are summarized in Table 2. The change of the composition led to more noticeable than that of the reaction time.

**Table 2:** Properties of lignin-PCL copolymers via grafting copolymerization using LMs through ROP of CL without solvent.

Sample	[CL]/[OH]^a^ ratio	Time^b^ (hr.)	M_n,L-PCL_ (gmol^−1^)^c^	M_w,L-PCL_ (gmol^−1^)^c^	MWD^c^	*n*^d^	M_w,PCL_ (gmol^−1^)^e^	α (%)^f^	T_id_ (2%)^g^ (°C)
LigPCL11	0.86	04	1,480	6,380	4.3	7	798	40	178
LigPCL13A	2.6	06	3,180	16,520	5.2	17	1,938	51	230
LigPCL13B	2.6	12	4,030	25,360	6.4	20	2,280	68	232
LigPCL13C	2.6	18	4,360	33,570	7.7	26	2,964	71	233

^a^[OH] of LM. ^b^Reaction time at 150 °C. ^c^Molecular weight of lignin-PCL copolymer was analyzed by GPC with polystyrene standards. THF was used as an eluent. ^d^The ‘*n*’ is degree of polymerization in PCL segments, and determined by ^1^H NMR end-group integration. ^e^The average molecular weight of a PCL segment per LM was calculated by ^1^H NMR end-group integration. ^f^The degree of grafting, **α** was calculated by {*f*_*LM*_ − *f*_*L_PCL*_}/*f*_*LM*_ of a LM, with *f*_*LM*_ denoting the functionality of an LM (15.3). The value of {*f*_*LM*_ − *f*_*L_PCL*_} suggests the number of graft PCL chains per LM. g) Initial deposition temperature (2%) was determined by thermogravimetric analyses (TGA).

**Figure 4:**
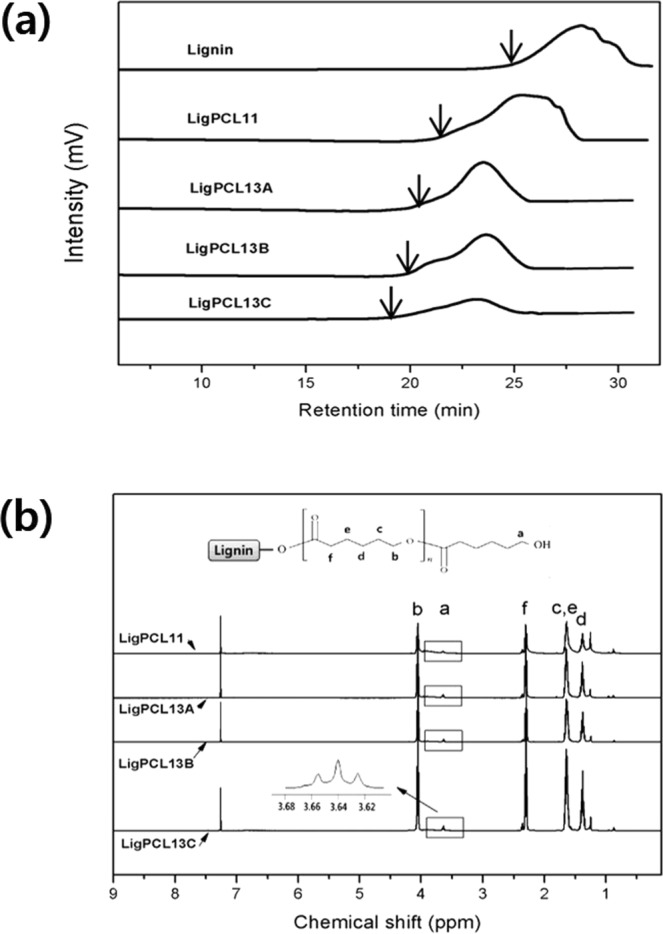
(**a**) GPC curves of pristine lignin and lignin-PCL copolymers. (**b**) ^1^H NMR spectrum of lignin-PCL copolymers in CDCl_3_(B) representing the structural change.

Figure 4(a) compares GPC chromatograms of the pristine lignin and lignin-PCL copolymers. The pristine lignin (LM) showed M_n,LM_ and M_w,LM_ at 970 and 1520 g mol^−1^, respectively. As shown for LigPCL11 and LigPCL13A, altering the [CL]/[OH] ratio from 0.80 to 2.60, the M_w,L-PCL_ increased from 6,380 to 16,520 g mol^−1^. Similar trend was obtained at [CL]/[OH] = 2.6, where the LigPCL13A and LigPCL13C values increased from 25,360 to 33,670 g mol^−1^ by varying the reaction time from 6 to 12 hours. Compared with LM, the molecular weight of LigPCL copolymers was enhanced. The change in molecular weight following altered reaction time and [CL]/[OH] ratio effectively improved the molecular weight of the copolymers. However, the increase in the molecular weight distribution of copolymers with MWD range of 4.3~7.7 may be pointing to poor control, probably due to irregular structure and complex reactivity of LM^32,33^. Despite of our efforts to provide pristine lignin with consistent and reliable reactivity, the inherent heterogeneity of pristine lignin could not be excluded completely^34–36^. The copolymer comprises both bulky LM and branched units, and the reactivity also varies due to the steric hindrance in the three-dimensional structure of lignin^36,37^. In addition to these unique characteristics of LM, the reason for broad MWD seems to coexist of different LMs with various PCL chains in copolymerization.

Figure 4(b) compares the ^1^H NMR spectra of lignin-PCL copolymers obtained under different reaction times. The copolymers exhibited resonances at 3.9~4.1 and 3.6~3.7 ppm, which were ascribed to the protons of the PCL repeating unit (I_b_) and the end-group of PCL segment (I_a_), respectively. ^1^H NMR end-group integration of these spectra can be used to calculate the degree of polymerization (*n*) in the PCL chain segments using the following formula:

2 $$n=(\frac{{I}_{b}}{{I}_{a}}+1)$$

where I_b_ and I_a_ correspond to the repeating and terminal ^1^H NMR methylene intensities of the PCL chain, respectively^19^. The *n* of PCL chain segments in lignin-PCL copolymers were 7 for ligPCL11 and 26 for ligPCL13C. The higher [CL]/[OH] ratio led to extended graft PCL chains in the lignin-PCL copolymers. When [CL]/[OH] was 2.60, the *n* also increased from 17 to 26 along the reaction time. The longer reaction time resulted in larger graft PCL chains in the lignin-PCL copolymers. Using *n* derived from ^1^H NMR, the molecular weight of PCL segment in lignin-PCL copolymer, M_w,PCL_ can be determined as follows:

3 $${{\rm{M}}}_{{\rm{w}},{\rm{PCL}}}=n{{\rm{M}}}_{{\rm{w}},{\rm{CL}}}$$

where M_w,CL_ denotes the molecular weight of CL monomer at 114 gmol^−1^. The molecular weight of lignin-PCL copolymer (M_w,L-PCL_) includes M_w,LM_ and the individual molecular weights of several grafting PCL chain segments (M_w,PCL_). The {*f*_*LM*_ − *f*_*L*-*PCL*_} represents the average number of PCL segments per LM and is calculated by the following equation:

4 $$\{{f}_{LM}-{f}_{L\_PCL}\}=\frac{{{\rm{M}}}_{{\rm{w}},{\rm{L}}\_{\rm{PCL}}}-{{\rm{M}}}_{{\rm{w}},{\rm{LM}}}}{{{\rm{M}}}_{{\rm{w}},{\rm{PCL}}}}$$

where M_w,L-PCL_ and M_w,LM_ are measured by GPC, and M_w,PCL_ is calculated. Further, the degree of grafting (*α*) is determined by the ratio of {*f*_*LM*_ − *f*_*L_PCL*_*}/f*_*LM*_ as shown in Eq. (1). Both experimental parameters, including [CL]/[OH] ratio and the reaction time, were systematically varied. When [CL]/[OH] is 0.86, α is 40%. α increased from 51%, 68%, and 71% with reaction time when [CL]/[OH] is 2.60. These are summarized in Table 2. Basis on the Fig. 2(c), the effect of [CL]/[OH] ratio and the reaction time in copolymers is shown by Fig. 2(d). Employing higher [CL]/[OH] ratio and longer reaction time produced lignin-PCL copolymers with higher molecular weight, with more PCL chains per a LM, and with a larger MWD. This result indicates that α refers to the active functionality of [OH] in LM. Eventually, by controlling the [CL]/[OH] ratio and the reaction time, the structure and Mw of LigPCL copolymers was modulated.

**Figure 5:**
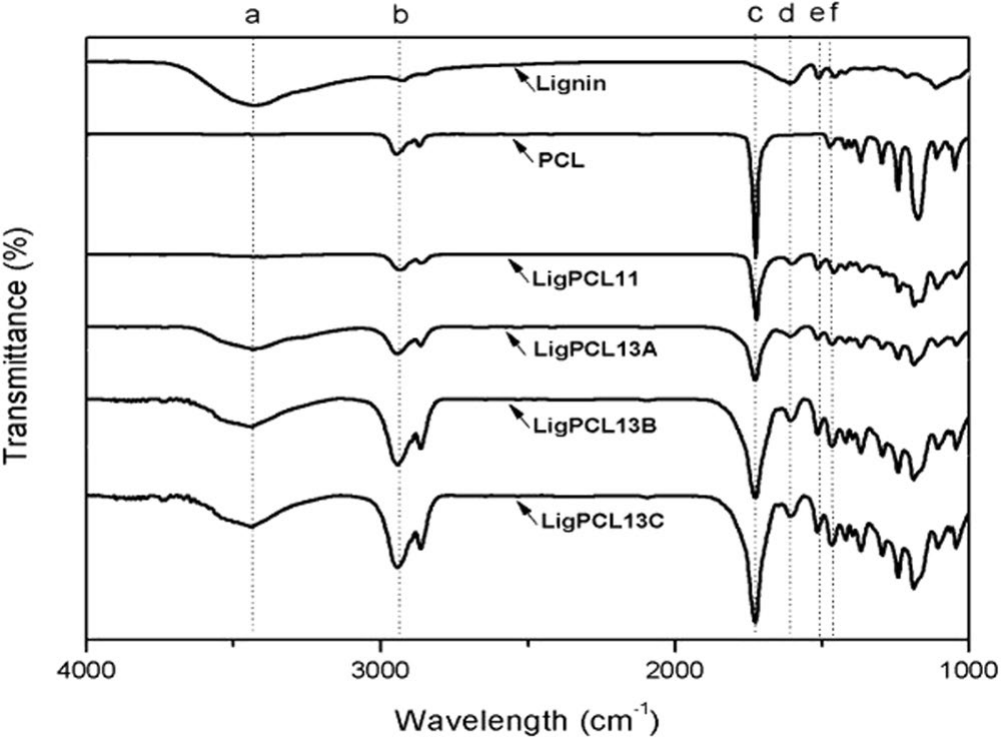
FT-IR spectra of pristine lignin, PCL and lignin-PCL copolymers.

Figure 5 compares the IR absorption spectra of the pristine lignin, PCL and lignin-PCL copolymers. FTIR spectroscopy is the most widely used technique in the functional group determination. The typical functional groups and the IR signal with the possible compounds are summarized in Table 3. A strong wide band near 3450 cm^−1^ was linked to the alcoholic and phenolic hydroxyl groups involved in hydrogen bonds. The absorption bands at 1603 cm^−1^ and 1503 cm^−1^ are aromatic ring vibrations, and the bands at 1460 cm^−1^ are assigned to stretching vibration of aromatic ring and deformation vibration of C-H bonds^23,38^. On the other hand, PCL spectrum show characteristic peaks of C=O stretching vibration at 1755 cm^−1^, and CH_2_ stretching vibrations at 2900 cm^−1^ ^39,40^. The lignin-PCL copolymer spectra obtained reveal an increased intensity of the ester C=O bond (1755 cm^−1^), correlated with a slight decrease in the broad OH signal (3450 cm^−1^) after copolymerization. In addition to, the presence of alkyl chain structure in PCL is shown by the small peak assigned to the CH_2_ stretch at 2900 cm^−1^. We confirmed the successful grafting of PCL chains on the LM.

**Table 3:** FT-IR spectra absorption bands of lignin, PCL and lignin-PCL copolymers^23,38–40^.

	Wavelength (cm^−1^)	Possible assignment
a	3450	O-H stretching vibrations
b	2900	C-H stretching vibration
c	1755	C=O stretching (ester bond)
d, e	1603 and 1504	C=C stretching of aromatic rings
f	1460	C-H deformation and aromatic ring vibration

### Compatibility analysis of lignin-PCL copolymers in organic solvents

**Figure 6:**
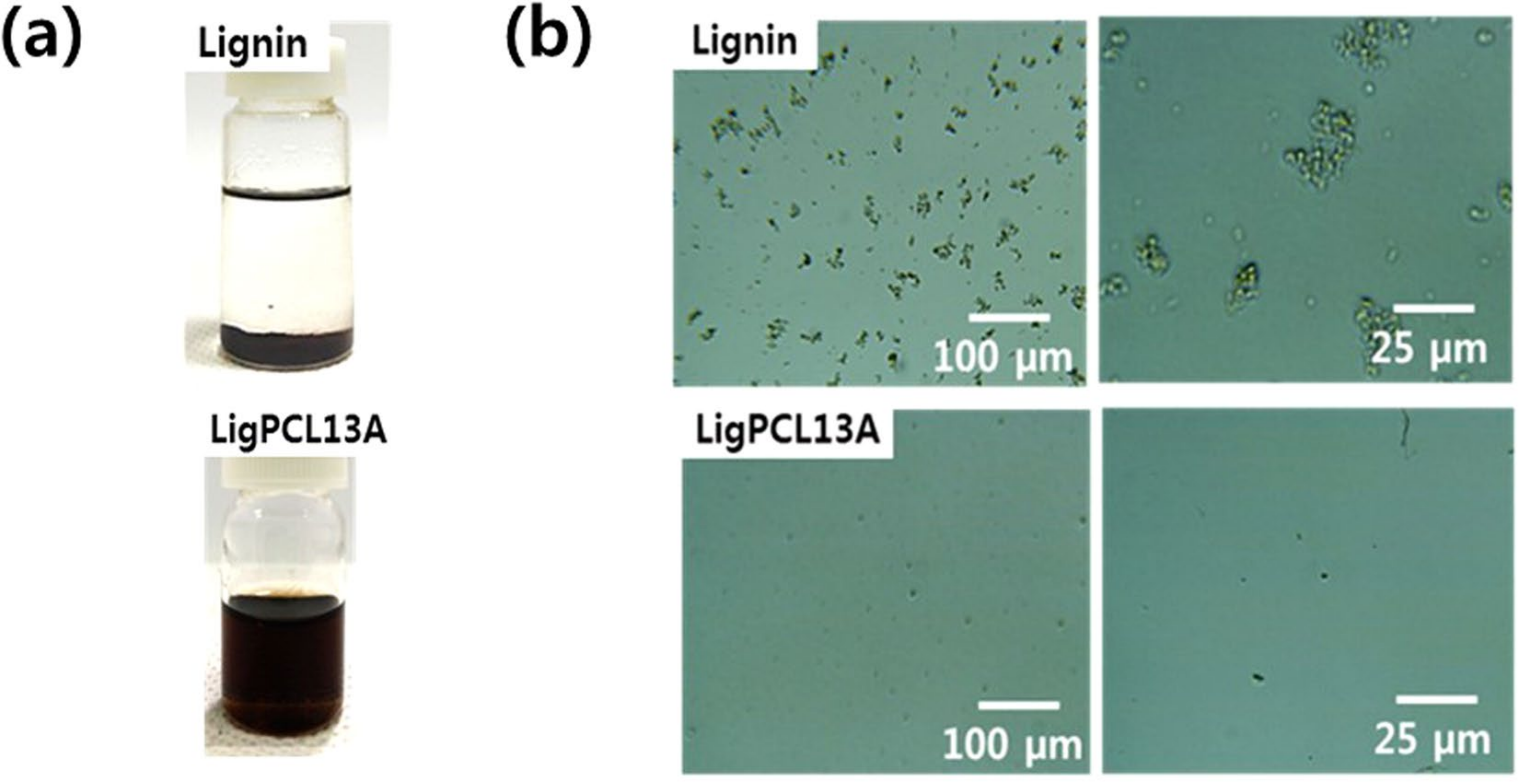
Photographic images of pristine lignin (up) and LigPCL13A (down) dispersed in toluene (**a**) optical microscopic images of pristine lignin (up) and LigPCL13A (down) in THF (**b**) demonstrating the altered solubility of the pristine lignin and the lignin-PCL copolymers.

Figure 6(a) illustrates similar (1 wt%) lignin and copolymers in toluene. The pristine lignin cannot be dissolved in nonpolar solvents but the lignin-PCL copolymers were soluble in toluene, clearly establishing the synthesis of lignin-PCL copolymer. Moreover, the optical morphologies of the pristine lignin and lignin-PCL copolymers in tetrahydrofuran (THF) are shown in Fig. 6(b), which reveal coagulation of the pristine lignin compared with complete dissolution of the lignin-PCL copolymer in the THF. The lignin aggregates owing to poor solubility in THF, whereas the lignin-PCL copolymer is soluble remarkably. The graft copolymerization of lignin-PCL copolymer was confirmed by of the altered solubility in toluene and THF.

### Thermal properties

**Figure 7:**
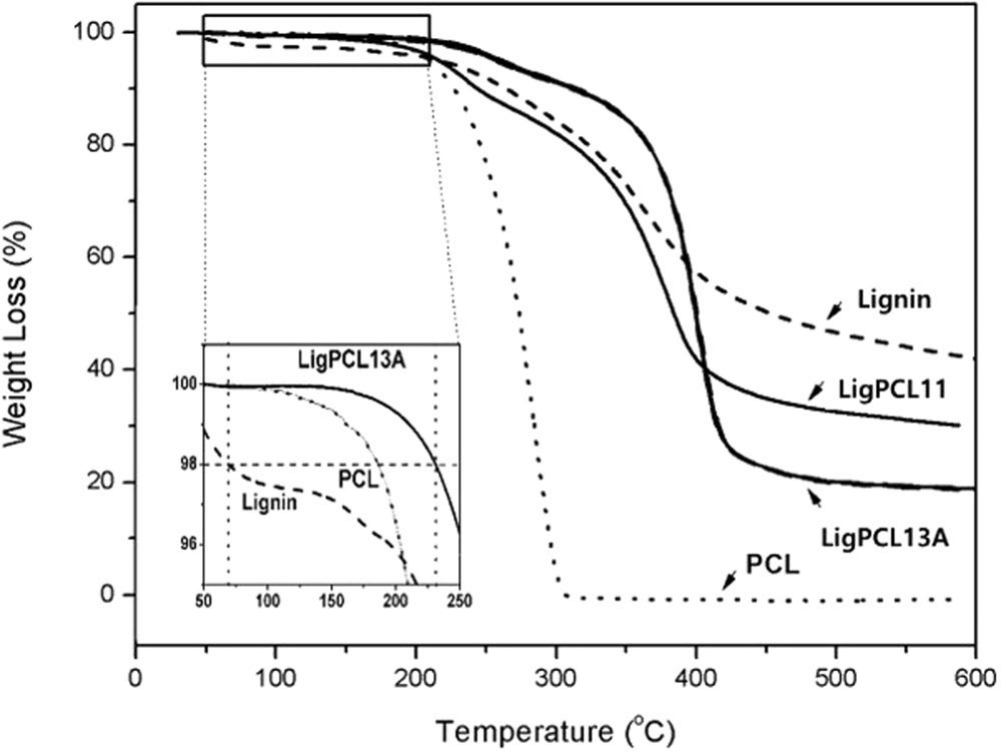
Thermogravimetric analyses of pristine lignin, PCL and lignin-PCL copolymers (LigPCL11 and LigPCL13A) in nitrogen from 30 °C to 600 °C at 10 °C min^−1^.

The thermal stability of the pristine lignin and the synthesized lignin-PCL copolymers are compared in Fig. 7. In order to quantitatively compare the thermal stability in the early stages of weight loss, the T_id_(2%) was defined as the temperature showing a 2% weight loss. T_id_(2%) is considered important because lignin usually emits an odor with accompanying fumes, which is indicated by the early-stage weight loss^6^. In this study, the T_id_(2%) of the pristine lignin was measured at 69 °C, while that of the synthesized lignin-PCL copolymers was 178 °C. Likewise, the weight loss of the pristine lignin at 200 °C was 4% compared with 0.6% in LigPCL13A. It clearly demonstrates that the synthetic lignin-PCL copolymer exhibited substantial improvement in thermal stability, which subsequently facilitated polymer composite melting at around 180 °C to 200 °C. The low molecular weight of lignin is related to the large molecular size of the copolymers. In addition, the hydrophilic nature of pristine lignin decreased substantially via condensation of hydroxyl groups in the lignin. The final TGA residue of the copolymers was lower than that of pristine lignin because of PCL inclusion in the copolymer chains.

### Melt process

**Figure 8:**
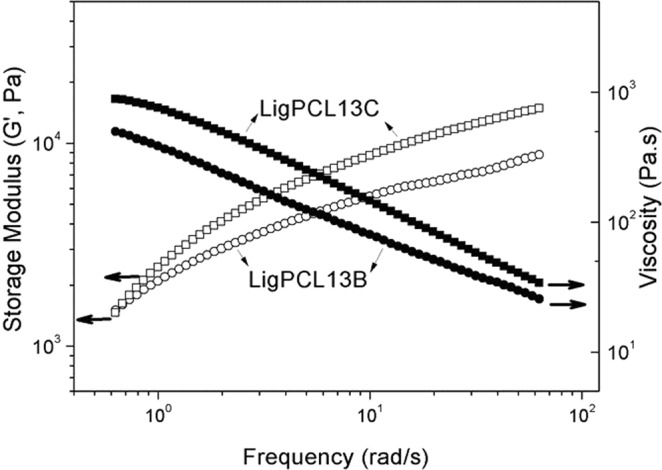
Storage modulus and viscosity angular frequency of LigPCL13B and LigPCL13C based on melt rheology at 180 °C.

Figure 8 illustrates the storage modulus (G′) and the viscosity (η) as a function of frequency of LigPCL13B and LigPCL13C at 180 °C. Storage modulus of LigPCL13B and LigPCL13C steadily increase with frequency. The viscosity of both lignin-PCL copolymers decreases with increasing frequency showing the typical shear-thinning behavior due to viscoelastic characteristics of polymers. The molecular weight of LigPCL13B and LigPCL13C is 25,360 g/mol and 33,560 g/mol, respectively. At low frequency LigPCL13C exhibits higher viscosity compared with LigPCL13B, which corresponds to the difference in M_w_ of the lignin-PCL copolymers. The thermoplastic characteristics of the synthesized lignin-PCL copolymers were established by measuring the viscosity.

**Figure 9:**
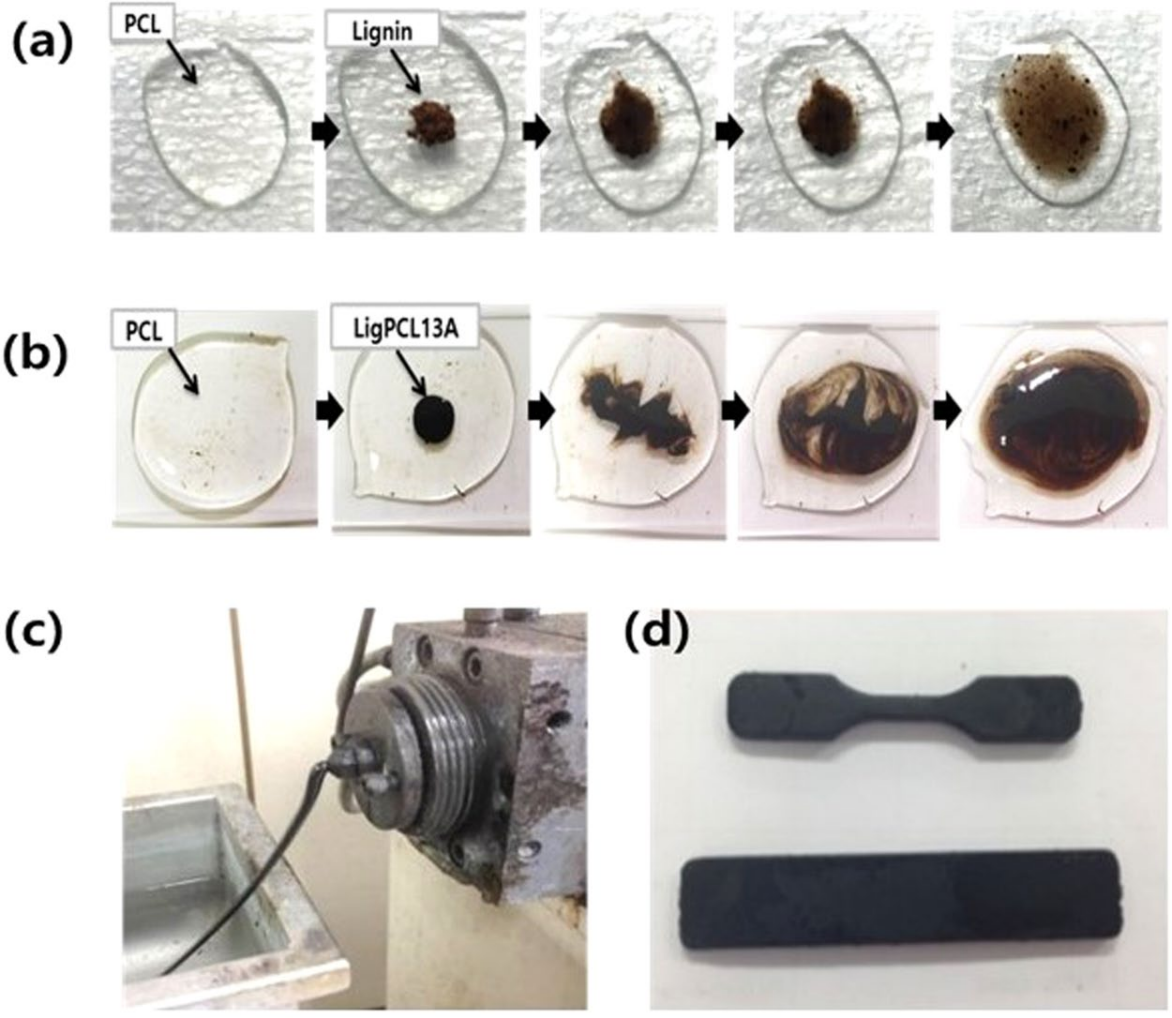
Photographic images of melt mixing of lignin and PCL at 150 °C (**a**) melt mixing of ligPCL13A and PCL at 150 °C (**b**) melt process of LigPCL13A at 180 °C by a twin-screw extruder (**c**) and samples with special shape of LigPCL13B obtained by injection moulding machine at 180 °C (**d**).

Figure 9 demonstrates melt mixing and processing characteristics of the synthetic lignin-PCL copolymers that are lacking in the pristine lignin. Figure 9(a) shows the dispersion of the pristine lignin in melt PCL at 150 °C, indicating poor miscibility. By contrast, Fig. 9(b) shows the well-mixed lignin-PCL copolymer with melt. Figure 9(c) shows molten LigPCL13A via extrusion at 180 °C. In Fig. 9(d), a dog-bone and a rectangular sample of LigPCL13B molded through injection molding machine demonstrating the thermal melting of the synthetic lignin-PCL copolymer.

## Conclusions

Lignin-PCL copolymers were successfully synthesized via ROP using CL as a reactive solvent. Depending on the [CL]/[OH] ratio and the reaction time, the graft polymerization with CL on a LM was controlled, and the molecular weight and the degree of grafting was modulated. Consequently, the synthetic lignin-PCL copolymers enhance the compatibility and thermal stability compared with pristine lignin and result in rheological behavior similar to that of thermoplastics without burning at high temperature. In particular, the lignin-PCL copolymers were melt-processed via bulk polymerization and provide new insights into this widely studied and commercially important polymer.

## Materials and Methods

### Materials

Lignin was extracted from black liquors using a method, which was developed by our laboratory and reported elsewhere^41^. The black liquor, an as-it-is waste of the kraft pulping process, was obtained from Moorim P&P, South Korea. Methanol (99%), ε-caprolactone (CL, 99%), dibutyltin dilaurate (DBTDL, 99%), sulfuric acid (H_2_SO_4_, 99%), tetrahydrofuran (THF), and dimethyl sulfoxide (DMSO-d_6_) were purchased from Sigma-Aldrich.

### Preparation of lignin acetylation for ^1^H-NMR analysis

The oven-dried lignin (1 g) was mixed with acetic anhydride-pyridine (3:10, v/v, and 13 mL) and vigorously stirred for 24 hours at room temperature^10^. The mixture was added dropwise in cold water and precipitated followed by centrifugation. The resulting solid product was thoroughly washed with DI water to remove the unreacted acetic anhydride and acetic acid byproducts. The product was dried overnight in an oven at 40 °C. ^1^H-NMR spectra of lignin and lignin-PCL copolymer were obtained by NMR spectroscopy (Varian UNITY INOVA 500).

### Preparation of lignin-PCL copolymers

The lignin was mixed in CL at 50 °C by protecting the nitrogen and stirring for 30 min. The DBTDL (0.5–1.0 wt% of lignin) was added slowly to the above mixture, which was heated to 150 °C for reaction times ranging from 4 to 18 h with mechanical stirring. When the reaction was finished, the product was cooled down to 25 °C, and the mixture was washed several times with a cool 95% methanol to remove impurities. During the reaction, the torque of a rotating spindle (130 rpm) was measured as a function of reaction time. Since the torque represents the altered viscosity or the molecular weight of the reacting mixture, the reaction time was determined *in*-*situ* by measuring the torque (see Fig. S2 in the Supplementary Information for the experimental setup). The reactor capacity was 1000 mL, and the spindle was installed with a torque sensor (RE-101, Well Corporation). Extrusion mixing of the lignin-PCL copolymers was carried out at 180–190 °C using a twin-screw extruder (BA-19, Bau-tech), and dumbbell (63.5 mm * 9.5 mm * 4 mm) and rectangular (70 mm * 12.7 mm * 4 mm) specimens were prepared using an injection molding machine (MM, Bau-tech) at 180 °C.

### Characterization

The infrared spectra of lignin, PCL, and the lignin-PCL copolymer were obtained using FT-IR spectroscopy (Bruker IFS-66/S). GPC (AT-400) was used to measure the molecular weight and its distribution in the lignin-PCL copolymers. TGA were performed in a nitrogen environment using a TG/DTA7300 system (Seiko instruments), by heating the specimens to 600 °C at a rate of 10 °C/min. The viscosity of these samples was analyzed in a dynamic mode of shearing on a rheometer ARES (TA Instrument) using a parallel plate geometry with 25 mm diameter. All experiments were carried out at 180 °C in the frequency range 0.5 < ω (rad.s^−1^) <50. Digital photographs of the sample and process were obtained using digital camera (Apple Inc.). The solubility of the pristine lignin and the lignin-PCL copolymers in THF was analyzed using an optical microscope (Nikon Eclipse Ni-E).

## Supplementary information

Supplementary info
