# Author Correction: Solvent-free bulk polymerization of lignin-polycaprolactone (PCL) copolymer and its thermoplastic characteristics

**DOI:** 10.1038/s41598-020-64444-z

**Published:** 2020-05-05

**Authors:** In-Kyung Park, Hanna Sun, Sung-Hoon Kim, Youngjun Kim, Go Eun Kim, Youngkwan Lee, Taesung Kim, Hyouk Ryeol Choi, Jonghwan Suhr, Jae-Do Nam

**Affiliations:** 1 Department of Polymer Science and Engineering, Sungkyunkwan University, Suwon, 16419 Republic of Korea; 2 Department of Energy Science, Sungkyunkwan University, Suwon, 16419 Republic of Korea; 3 School of chemical engineering, Sungkyunkwan University, Suwon, 16419 Republic of Korea; 4 School of Mechanical engineering, Sungkyunkwan University, Suwon, 16419 Republic of Korea

Correction to: *Scientific Reports* 10.1038/s41598-019-43296-2, published online 07 May 2019

The original version of this Article contained a typographical error in the spelling of the author Hyouk Ryeol Choi, which was incorrectly given as Hyun-Ryeol Choi. This has now been corrected in the PDF and HTML versions of the Article, and in the accompanying Supplementary Information file.

