# Supplementary material for: Solvent-free bulk polymerization of lignin-polycaprolactone (PCL) copolymer and its thermoplastic characteristics
Source: Sci Rep. 2019 May 7;9:7033. doi: 10.1038/s41598-019-43296-2 (PMC6504852; doi:10.1038/s41598-019-43296-2)
Supplement: Supplementary file 1 — Supplementary info [file 41598_2019_43296_MOESM1_ESM.docx]

Supplementary Information for

**Solvent-free bulk polymerization of lignin-polycaprolactone (PCL) copolymer and its thermoplastic characteristics**

**In-Kyung Park, Hanna Sun_,_ Sung-Hoon Kim ,Youngjun Kim, Go Eun Kim, Youngkwan Lee, Taesung Kim, Hyouk-Ryeol Choi, Jonghwan Suhr and Jae-Do Nam**


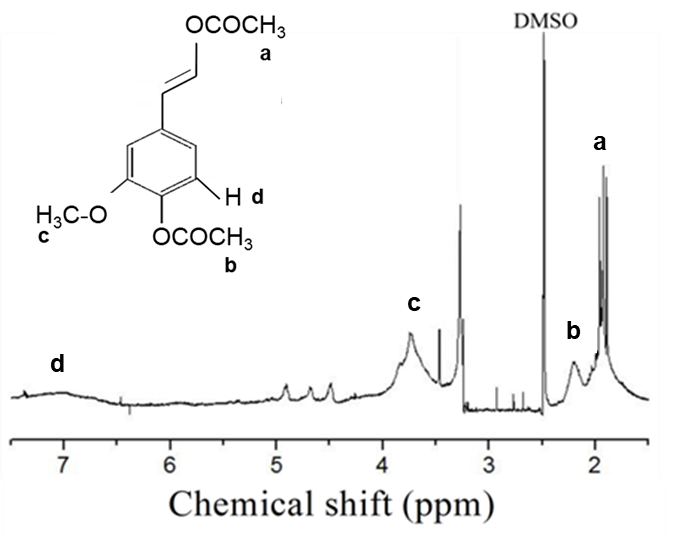


**Figure S1. ^1^**H-NMR spectrum of acetylated kraft lignin.


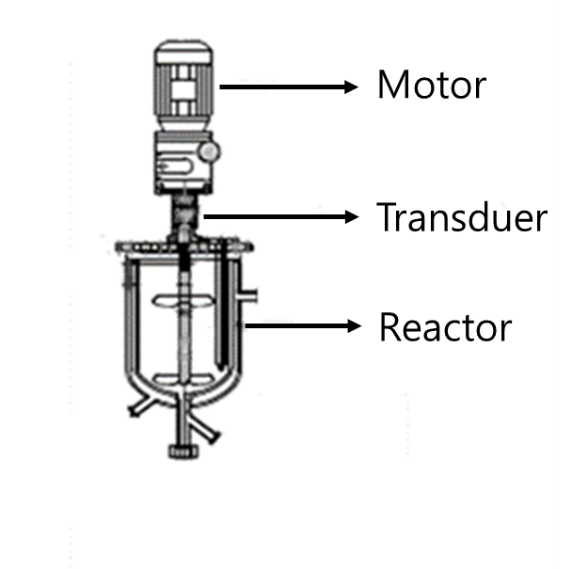


**Figure S2.** Reactor mounted with the rotary torque transducer
